# Inter-personal motor interaction is facilitated by hand pairing

**DOI:** 10.1038/s41598-021-04595-9

**Published:** 2022-01-11

**Authors:** Keivan Mojtahedi, Kimia Kiani, Marco Santello, Qiushi Fu

**Affiliations:** 1 School of Biological and Health Systems Engineering, Arizona State University, Tempe, AZ 85287-9709 USA; 2 Mechanical and Aerospace Engineering, University of Central Florida, Orlando, FL 32816 USA; 3 Present Address: Dexcom, San Diego, CA 92121 USA

**Keywords:** Human behaviour, Motor control, Social behaviour

## Abstract

The extent to which hand dominance may influence how each agent contributes to inter-personal coordination remains unknown. In the present study, right-handed human participants performed object balancing tasks either in dyadic conditions with each agent using one hand (left or right), or in bimanual conditions where each agent performed the task individually with both hands. We found that object load was shared between two hands more asymmetrically in dyadic than single-agent conditions. However, hand dominance did not influence how two hands shared the object load. In contrast, hand dominance was a major factor in modulating hand vertical movement speed. Furthermore, the magnitude of internal force produced by two hands against each other correlated with the synchrony between the two hands’ movement in dyads. This finding supports the important role of internal force in haptic communication. Importantly, both internal force and movement synchrony were affected by hand dominance of the paired participants. Overall, these results demonstrate, for the first time, that pairing of one dominant and one non-dominant hand may promote asymmetrical roles within a dyad during joint physical interactions. This appears to enable the agent using the dominant hand to actively maintain effective haptic communication and task performance.

## Introduction

Physically-coupled motor actions between two agents play an important role in a wide variety of activities, including hand-shaking, dancing with a partner, moving heavy objects, or assisting patients during physical rehabilitation. Such interactions often involve coordination of actions with minimal or no verbal communication. Despite the prevalence of physical coupling in daily activities, joint actions have been traditionally examined using tasks that are mediated only by visual and/or auditory coupling, with the focus being on the underlying sensorimotor and cognitive processes^1,2^. Several recent studies have started to examine how inter-personal coordination can be mediated by haptic coupling. It has been demonstrated that effect of haptic feedback on the performance of joint actions could be influenced by many factors, e.g., the stiffness of the physical coupling^3^, spatial configuration of the interacting partners^4^, and skill levels of individual agents^5,6^. Furthermore, it was found that physically-coupled dyads could contribute to the task asymmetrically^7–9^. However, most of these studies have usually used a single dyadic configuration. Some studies used a dominant and a non-dominant hand, e.g., side-by-side configuration ^10,11^. Alternatively, other studies have focused on dominant-dominant pairing^5,8^.

**Figure 1:**
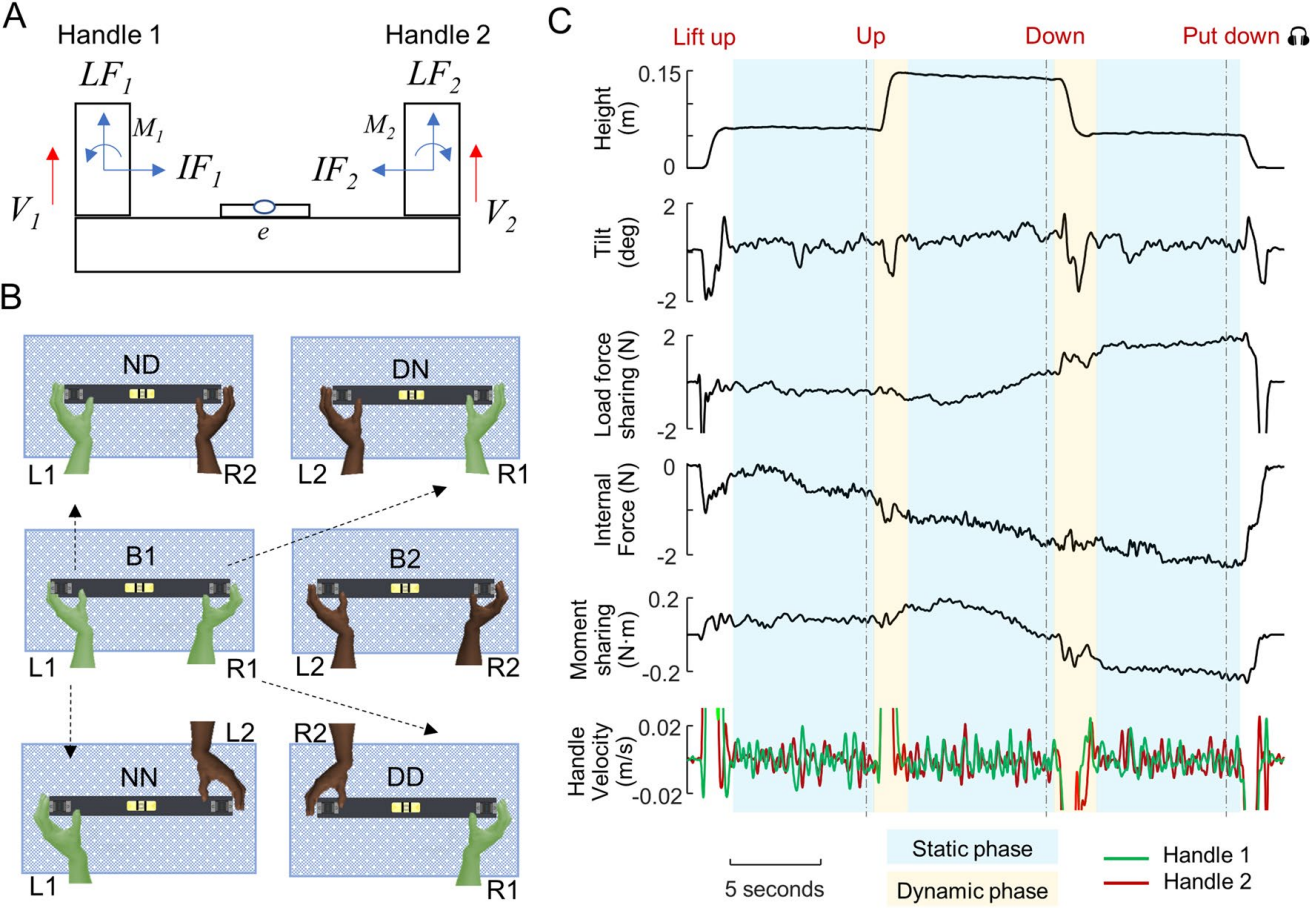
Experimental design and definitions of variables related to coordination. (**A**) We defined behavioral variables with respect to the object to be balanced by two hands. Each hand (from one or two participants) grasped one handle with thumb on the inner portion of the handle. *e* denotes the tilt angle as indicated by a bubble level. *LF*_*i*_, *IF*_*i*_, *M*_*i*_, and *V*_*i*_ are load forces, normal forces, in-hand moments, and handle vertical velocity measured at handle *i* (1 or 2), respectively. The direction of the arrows represents posititive directions. (**B**) Top view of six different task conditions: bimanual solo (B1 and B2), dyadic different-hand (DN and ND), and dyadic same-hand (DD and NN, respectively). The first and second letters of these acronyms denote the hands used by the participants from B1 and B2, respectively. (**C**) From top to bottom: object vertical position, object tilt (performance error, *e*), internal force, load force sharing, in-hand moment sharing, and handle vertical velocities. Dynamic and static phases are denoted by yellow and blue shading, respectively. Data are from one subject pair (DD condition). The timeline of auditory cues is denoted by vertical dashed lines.

In a recent study, we recruited 72 participants whom were randomly paired to form 36 dyads to perform physically-coupled joint actions with several different hand pairing scenarios^12^. Each dyad performed six blocks of an object balancing tasks (Fig. 1A). In two baseline bimanual blocks, each participant held the object with both hands and performed the task individually. The other four blocks were performed by dyads with each participant using either the dominant or non-dominant hand (Fig. 1B). We focused on how task performance, i.e., minimizing object tilt, was affected by hand pairing in dyads. We found that side-by-side pairing of one dominant and one non-dominant hand resulted in better performance than face-to-face pairing of two dominant or two non-dominant hands. However, we did not address the extent to which hand dominance and pairing configuration might have altered the underlying motor coordination strategies between two agents, i.e., how do two hands contribute in the joint performance of the task?

In the present study we addressed this gap by quantifying the motor coordination patterns with kinetic and kinematic data recorded in our previous study. We are particularly interested in four aspects of motor coordination between participating hands. In the force domain, we quantified (1) how force was shared vertically to support object weight, and (2) the magnitude of horizontal forces exerted against each other. In the movement domain, we quantified (3) the vertical movement speed of each hand to maintain object balance, and (4) the temporal synchrony between the vertical hand movements. The vertical force sharing and vertical movement speed were used to assess the contribution symmetry between two hands. These metrics denote the extent to which each hand takes different roles in balancing the object while holding it in the air. In contrast, the horizontal opposing force and vertical movement synchrony were used as a proxy to evaluate the efficacy of the haptic communication. We hypothesized that hand dominance of the paired individuals performing our task would modulate all these behavioral aspects across dyadic conditions. There is extensive evidence showing advantages of the dominant or non-dominant limb in different types of motor tasks. It has been suggested that the neural mechanisms underlying sensorimotor control of the upper limb are lateralized^13^, which would lead to better performance of the dominant hand in tasks that rely on predictive control and better performance of the non-dominant hand in tasks that favors impedance control. Additionally, it has been observed that the dominant and non-dominant hand may be characterized by different properties, i.e., strength and motor noise which, in turn, may affect the cost functions driving the contribution of each hand during bimanual coordination^14^. Therefore, we expected that, when pairing one dominant and one non-dominant hand, the non-dominant one would take a more passive role due to its advantage in impedance control by carrying more weight and moving slower than the non-dominant one. We also expected that pairing of two non-dominant hands would be associated with the weakest haptic communication, i.e., smallest horizontal forces and least movement synchrony, due to weaker predictive control capability.

## Results

In our object balancing task, participants were asked to prioritize keeping the bubble at the center of the spirit level attached to the object (Fig. 1A) while following auditory cues to either move the object vertically between two target heights or hold it (Fig. 1C). The transition time between two heights (i.e., dynamic phase, Fig. 1C) was relative short, and here we only focus on the static phases (24 segments in each experimental condition, which were then averaged within each condition). There were four dyadic conditions and we define them with respect to one of the two participants in each dyad (Fig. 1B) who performed B1 bimanual condition (chosen randomly before the experiments started). There were two conditions in which two participants used different hands by sitting side-by-side (dominant-non dominant; DN and ND, respectively), whereas the other two conditions required two participants to use same hands by sitting face-to-face (DD and NN) (Fig. 1B). We used this experimental design to test the effect of hand dominance nested in a two-level pairing configuration factor.

### Load force sharing

To overcome gravity and keep the object static in the air, participants had to produce forces on the handles in the vertical direction (i.e., load force; *LF*_*1*_ and *LF*_*2*_ in Fig. 1A) whose sum had to match the weight of the object. However, there are infinite feasible combinations of two load forces that would fulfill this task constraint. It is important to note that a difference between the load forces exerted on two handles would result in a destabilizing moment on the object. This moment can be compensated by the difference between the in-hand moment produced by each hand on the handles (*M*_*1*_*, M*_*2*_ in Fig. 1A) to meet the balance requirement. Our analysis only focused on the *load force sharing* pattern, defined as the difference between two load forces, to quantify the asymmetry in the contribution to overcome gravity (with zero being symmetrical sharing). This approach was motivated by the fact that in-hand moment sharing patterns were highly correlated with load force sharing due to the balance requirement (see “Methods” and Supplementary Fig. 1).

**Figure 2:**
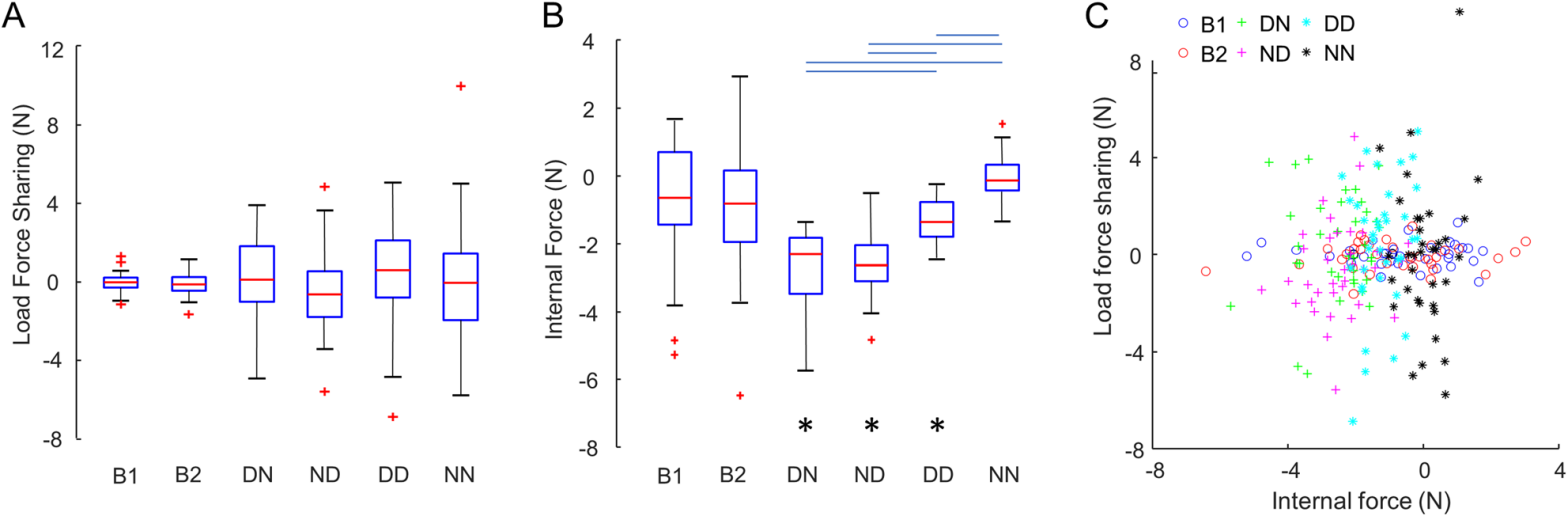
Force coordination in object balancing tasks. (**A**) Sharing of load forces between two handles. Boxplots show median values as red lines, with 25 and 75 percentiles depicted as blue boxes. The whiskers extend to most extreme points that are not potential outliers (i.e., red crosses). (**B**) Internal force. Conditions joined by the two ends of the blue horizontal lines are statistically different. The bottom asterisks represent significanct difference from zero. (**C**) Distribution of force coordination strategies. Each data point represents the average force pattern of a participant (bimanual conditions) or a dyad (dyadic conditions).

For bimanual conditions, this metric was computed by subtracting the load force exerted by the left hand from the load force exerted by the right hand. We found that B1 and B2 had similar load force sharing (t-test, p = 0.616) with no hand dominance-related asymmetry in intra-personal coordination (one-sample t-test, p = 0.374). For dyadic conditions, the load force sharing metric was computed by substracting the load force exerted by the participant in B2 from the load force exerted by the participant in B1. This definition was used because there was not always a right and a left hand involved, i.e., in DD and NN conditions. Furthermore, this consistent reference to B1 participants enabled us to test whether dyad-specfic load force sharing patterns exist across conditions using a random intercept in a linear mixed model (see “Methods”). We found that hand dominance, pairing configuration or their interaction had no significant effect on load force sharing (p = 0.506, 0.873 and 0.309, respectively). These results suggest that hand dominance does not play a role in the sharing of load force in neither bimanual or dyadic interactions. However, we found a significant random effect (p < 0.001), which indicates that one participant tends to always carry more load than the other one regardless of the hand used or spatial pairing configuration of the agents. We also found that, although all individuals and dyads had a mean load force sharing of about zero within each condition, dyads were characterized by larger within-condition (inter-dyad) variance across different dyads than the within-condition (inter-subject) variance across inidividuals performing bimanually (Fig. 2A). Note that this wide range of dyad-specific load force sharing patterns indicates that dyads were more likely to adopt an asymmetrical load force sharing than when either agent used two hands in the bimanual conditions. We statistically evaluated this observation by comparing the absolute deviations from the condition mean using Wilcoxon Signed Ranks tests. We found that the load force sharing variabilty was significantly smaller in the bimanual than dyadic conditions (p < 0.05), whereas no difference was found between the dyadic conditions (Supplementary Fig. 2).

### Internal force

Although subjects were not given explicit instructions about controlling the handle’s horizontal position, the object was kept stationary horizontally most of the time. While two hands acted against gravity in the vertical direction, they could also act against each other in the horizontal direction, and these forces must be exerted in equal magnitude but opposition directions (*IF*_*1*_ and *IF*_*2*_; Fig. 1A). We define this force as the internal force (see “Methods”). There are infinite number of feasible values this internal force can take as long as two sides equally oppose each other. For bimanual conditions, we found that B1 and B2 had similar internal force (t-test, p = 0.718). In contrast, we found different magnitudes of internal force in dyadic conditions (Fig. 2B). A linear mixed model revealed a significant pair-wise random effect (p < 0.001), suggesting a pair-specific preference of internal force magnitude. There was also a significant interaction between hand dominance and hand pairing configuration (p < 0.001). Post-hoc t-tests showed that dyads used similar outward internal force in both different-hand conditions (mean ± S.E.: 2.64 ± 0.18 N and 2.56 ± 0.16 N for DN and ND conditions, respectively), which were larger than other conditions (p < 0.05). Specifically, the internal force was significantly smaller in the DD condition (1.28 ± 0.11 N outward), and smallest in the NN condition (0.03 ± 0.11 N inward, indifferent from zero). These results suggest that, in addition to pair-wise preference of internal force, both hand dominance and pairing configuration play important roles in determining internal force.

We also observed that bimanual conditions exhibited a much larger range of internal force between individual participants than across dyads. Note that load force sharing was characterized by the opposite pattern (Fig. 2C). This observation was again evaluated by comparing the absolute deviations from the condition mean (Supplementary Fig. 3). Wilcoxon Signed Ranks test showed that bimanual conditions had the largest within-condition (inter-subject) variability, whereas same-hand conditions (DD and NN) had the smallest within-condition (inter-dyad) variability, respectively.

### Handle vertical velocity

**Figure 3:**
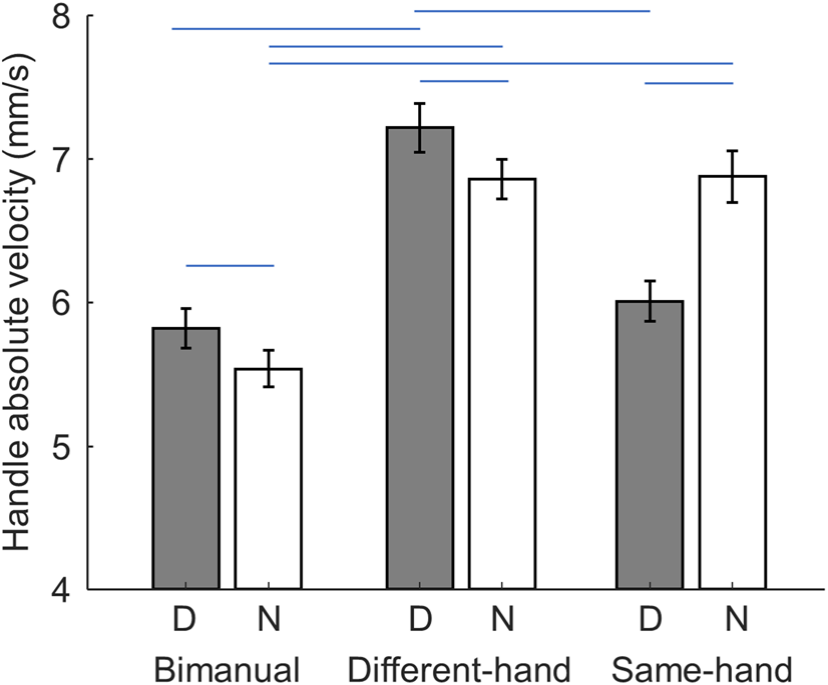
Handle movement velocity. The mean absolute value of handle vertical velocity are shown for each hand (mean ± S.E.; Dominant: D, Non-dominant: N). Conditions joined by the two ends of the blue horizontal lines are statistically different.

Although the task requires participants to maintain the height and balance during static holding, subtle movement of the hands still occurred. This could have been due to intentional change of load force, motor noise, or corrective actions generated to compensate the first two sources of variance. Regardless of the source of movement generation, we assessed the kinematic contribution of each hand by computing the amplitude of handle vertical movement velocity which represents how each hand actively participated in the joint action. Note that, unlike the load force sharing, there are no explicit mechanical or task constraints associated with the movement magnitude of two hands. Therefore, we can quantify the vertical velocity of the two hands separately. We first compared the vertical velocity of the right and left hands across all participants in bimanual conditions. We found no difference between B1 and B2, but also found that the dominant hand moved significantly faster than the non-dominant hand (5.82 ± 0.14 mm/s and 5.54 ± 0.13 mm/s, respectively; main effect of Hand, mixed two-way ANOVA, p < 0.001; Fig. 3). For dyadic conditions, a linear mixed model revealed a significant random-effect (p < 0.001) that indicates participant-specific preferences of hand movement velocity across conditions. More importantly, we found a significant interaction between hand dominance and pairing configuration (p < 0.001). Post-hoc comparisons showed that the movement velocity of the non-dominant hand was similar in both same- and different-hand conditions (6.86 ± 0.14 mm/s and 6.87 ± 0.18 mm/s, respectively), which were both faster than movement velocity of the non-dominant hand in bimanual conditions (p < 0.001). Interestingly, the dominant hand moved significantly faster (7.21 ± 0.17 mm/s) than the non-dominant hand (6.87 ± 0.18 mm/s) when they were paired in different-hand conditions (p = 0.007). In contrast, the dominant hand moved significantly slower (6.01 ± 0.14 mm/s) than the non-dominant hand (6.86 ± 0.14 mm/s) when they were engaged in the same-hand condition (p = 0.003). Moreover, the vertical velocity of the dominant hand was similar in bimanual and same-hand conditions. These results suggest that the dominant hand moved faster than the non-dominant hand in both Bimanual and side-by-side conditions in which one dominant hand cooperates with a non-dominant hand. Furthermore, the behavior of the dominant hand was affected by the pairing configuration, whereas the non-dominant hand was not.

### Delay between handle movements

**Figure 4:**
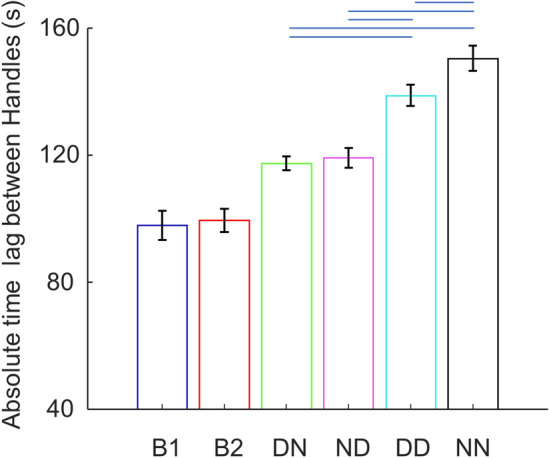
Time lag between handle movement velocities. Absolute time lag between handle vertical movement velocities identified by peak positive cross-correlation. Conditions joined by the twol ends of the blue horizontal lines are statistically different.

To assess the temporal synchrony of the vertical movement between two hands, we quantified the absolute time lag between the movement. This was accomplished by identifying the peak positive correlation in the normalized cross-correlation function between the vertical velocity profiles within each static phase segment. For bimanual conditions, we found no difference between B1 and B2 (97.89 ± 4.63 ms and 99.43 ± 3.64 ms, respectively). In contrast, for dyadic conditions we found a pattern similar to internal force (Fig. 4). Specifically, a linear mixed model again revealed a significant random effect (p < 0.001), suggesting pair-specific lags across conditions. Furthermore, we found a significant interaction between hand dominance and pairing configuration (p = 0.042). Post-hoc comparisons showed that dyads had a similar time delay between two hands in both different-hand conditions (117.41 ± 2.15 ms and 119.08 ± 3.15 ms for DN and ND, respectively), which was longer than bimanual condition B1 (p < 0.001). Moreover, the same-hand with dominant hands condition (DD) were characterized by a greater delay (138.78 ± 3.37 ms) than both-different hands condition (p < 0.001), whereas the same-hand non-dominant hands condition (NN) had the longest delay (150.48 ± 3.95 ms; p < 0.001 compared to DD). Interestingly, by comparing Fig. 4 and Fig. 2B we can observe a relation between the internal force metric and the time lag between hand movement metric in dyadic conditions. This was statistically confirmed by a significant correlation between these two metrics over all dyadic conditions and pairs (144 samples, Pearson’s r = − 0.514, p < 0.001; Supplementary Fig. 4). Furthermore, correlation analysis within each condition also supported this relation (DN: r = − 0.423, p = 0.010; ND: r = − 0.449, p = 0.006; DD: r = − 0.797, p < 0.001; NN: r = –0.406, p = 0.014). In contrast, we did not find a significant correlation between these two metrics in bimanual conditions.

## Discussion

Previous research has reported asymmetrical division of roles in a collaborative physical tasks that require attainment of a common goal. In a fast disk rotation task, some dyads implemented an ‘acceleration/deceleration’ strategy such that each agent focused on a phase of the task^7^. Similar phase difference were also found in joint wrist movement tracking tasks^8^. However, the overall effort spent by each agent was generally equal (but with different timing). A follow-up study using the wrist movement tracking task suggests that the partner with worse solo unimanual performance could improve at the cost of the better partner’s effort, but not at the cost of the better partner’s performance^3^. In the present study, we examine role asymmetry with two metrics in the kinetic (load force sharing) and kinematic (movement velocity) domains.

We first examined how force in the vertical direction may be shared by two hands to sustain the object weight. Previous studies in bimanual isometric force production have shown that the distribution of forces between two hands may be determined by quadratic cost functions associated with the effort and variability of each hand, which could vary across force levels^15^ and force directions^14^. In the present study, we found that the sharing of load forces between two hands were mostly even in bimanual conditions. Such symmetry indicates that the two arms share similar motor costs within each individual, i.e., no effect of hand dominance. This could be due to the fact that our task requires relatively low forces. In contrast, dyads were more likely to share the total load force asymmetrically (Fig. 2A). This means that one of the agents needed to produce higher level of load force per limb than he/she did in bimanual conditions. Moreover, the asymmetry in load force also required in-hand moment to compensate, whereas symmetrical load force sharing (as in bimanual conditions) does not. In fact, both agent in a dyad tend to produce higher level in-hand moment in dyad conditions than they did in bimanual conditions, as they deviate from symmetric load sharing (Supplementary Fig. 5). Therefore, dyads were much more likely to perform our task with larger overall effort than individual agents in bimanual conditions. From an optimization perspective, one individual’s objective of minimizing his/her own effort and variance (both are lower at lower force magnitudes) could conflict with the same objective of the partner. A global optimal solution (i.e., symmetrical load force sharing as found in the bimanual condition) can be achieved only if each agent takes the other agent’s cost function fully into consideration. However, the extent to which one agent is willing and/or capable to do so is difficult to assess and could be determined by multiple factors on a dyad-to-dyad basis. Our data rule out the contribution of hand dominance and pairing configuration because the load force sharing asymmetry persisted across different dyadic hand pairings. In other words, the agent who sustained a greater portion of the object’s weight tended to do so across all hand pairings with the same partner. In future studies, we plan to examine the following two factors that may predict the inter-personal sharing of effort in dyadic actions. First, the strength of paired individuals, such as grip and arm strength, should be assessed. This is because a stronger individual may perceive the same force as equivalent to lower effort, thus resulting in carrying more load. Second, the cognitive aspects of the paired individuals should also be evaluated with questionaires as it may determine their inner drive to team work. An example is the autism-spectrum quotient (AQ)^16^, which can be used to quantify social skills by identifying autistic traits. It has been demonstrated that AQ scores can predict the temporal lead-follow relation in dyads performing rhythmic movements^17^. Another tool is the interpersonal reactivity index (IRI)^18^, which can evaluate cognitive empathy defined as the tendency to adopt another’s view point. A recent study showed that empathic-perspective taking can help synchronizing actions in a joint music-making task^19^.

Another metric we investigated in relation to role asymmetry was the speed of the vertical handle movement. In contrast to load force sharing, we observed a clear effect of hand dominance in this kinematic metric, revealing that the dominant hand moved faster in bimanual conditions as well as in different-hand dyadic conditions (Fig. 3). Hand dominance has been mostly studied in motor tasks performed by a single individual instead of dyads. One popular theory for motor lateralization is the dynamic dominance model ^13^. This model posits that the dominant hemisphere is specialized for predictive control with good estimation of the limb dynamics, whereas the non-dominant hemisphere is specialized for impedance control that is based on feedback loops. A recent study used a fast bimanual spring strectching task and showed that the non-dominant (left) hand was better at stabilizing the end of the spring that needed to be held in place, whereas the dominant (right) hand was more precise at reaching with the other end of the spring^20^. Based on this framework, we speculate that the faster movement of the dominant hand in our bimanual and different-hand conditions may represent its more active contribution, whereas the non-dominant hand would have behaved more passively. Moreover, we consider it unlikely that motor noise can account for these results. This is because the motor noise of the dominant hand is expected to be lower than that of the non-dominant hand at the same force level^14^, and the dominant hand did not consistently exert more force than the non-dominant hand.

An interesting result regarding movement velocity was that the dominant hands moved much slower when paired together relative to the non-dominant hand pairing. This reversed contrast was mostly caused by the slower movement of the dominant hand whereas the non-dominant hand’s velocity was mostly invariant across different dyadic conditions. In fact, the movement velocity of the dominant hand in the same-hand condition was similar to that in bimanual conditions. Although it is difficult to separate the source of these movement variations, it can be inferred that the dominant hand was able to adjust its behavior in response to the dyadic pairing scenario whereas the non-dominant hand could not. This finding further supports the notion that the non-dominant hand acted in a more passive fashion than the dominant hand in our dyadic task. A potential confound in our study is that the subjects’ spatial configuration allowed the paired individuals to see each other’s face during same-hand but not different-hand conditions. Although our task requires high accuracy and full attention to the bubble level, it is still possible that dyads could have seen the facial expressions and gaze direction of the partner. Therefore, these subtle visual cues could have interfered with the underlying motor coordination in face-to-face conditions^4^. Future studies should consider blocking the view of partner’s faces.

We note that hand dominance plays a role not only in determining the kinematic contribution of the effectors in our task, but also in modulating the efficacy of haptic communication, as indicated by internal force (Fig. 2B) and movement synchrony (Fig. 4) metrics. It has been speculated that the internal force in inter-personal coordination could be a means to form a ‘haptic channel’ that facilitates haptic communication for enabling estimation of the partner’s goal^7,11,21–23^, such that it could compensate for lack of complete information about the other agent’s effector. A recent study found that the temporal structure of haptic signals is much more complex in dyads than that found in bimanual coordination^24^. Our finding of strong correlation between internal force and inter-agent movement delay provides additional evidence in support of the above theory, suggesting better action/perception coupling via haptic communication is associated with larger inter-personal forces. Note that such correlation is less likely to be explained by the mechanical stabilizing effect of increased stiffness caused by higher internal force. This is because internal force was measured in the horizontal direction whereas the movement was measured in the vertical direction. Additionally, these two metrics were not correlated within bimanual conditions in which haptic communication was not strictly necessary as the brain has complete information of both effectors. It is possible that efferent copies of the motor command to both limb are shared through direct inter-hemispheric communication to facilitate the prediction of the sensory consequences of bimanual actions^25,26^, thus leading to shorter delays between two hands of the same agent. In contrast, the time delay between two paired individuals could be caused by the sensorimotor delay involved in the continues monitoring and probing of the other one’s action through the haptic channel.

Importantly, we show that both hand dominance and pairing configuration modulate the haptic channel efficacy in dyadic conditions. To interpret this finding, we propose that pairing hands of different dominance enables role specialization to maintain a effective haptic channel. The dominant hand primarily drives the haptic communication through predictive control mechanisms whereas the non-dominant hand supports the haptic channel reactively through impedance control. When two non-dominant hands were paired, the haptic communication could be significantly weakened due to overall weaker predictive control. In contrast, when two dominant hands were paired, interference might have occurred as both hands were trying to actively predict the consequnces of their own actions and, at the same time, the consequences of the action of the other one. There are several other factors that may also influence the haptic communication in our tasks, considering the pair-wise random effect found in our mixed model in addition to the hand dominance effect. Similar to what we have speculated for the pair-wise preference of load force contribution asymmetry, both physical and cognitive factors could play a role in dyadic haptic communication. Although the non-dominant arm is known to be weaker than the dominant one^27^, it is unlikely that this strength difference was underlying the hand dominance effect we observed. If inter-limb strength difference had played a significant role, pairing of two dominant hands should have resulted in the largest internal force. Nevertheless, inter-personal strength differences may determine the overall preference for internal force, e.g., a stronger dyad may choose to exert a higher magnitude of internal forces. Social skills and personality could also determine the overall willingness to communicate during coordination. Therefore, future studies should use questionaires, such as above mentioned AQ and IRI, for further investigation.

In sum, our results reveal for the first time that pairing of one dominant and one non-dominant hand may promote an asymmetrical role assignment within a dyad. Such asymmetry enables the agent using the dominant hand to actively drive the dyadic interactions and maintain effective haptic communication. Future studies combining the present experimental task and neuroimaging techniques are needed to identify the cortical and sub-cortical networks involved in joint actions with different hand pairings.

## Methods

This paper focuses on analyses of force and movement data collected for a previously published study ^12^. We recruited 72 right-handed participants (age: 19–31 years, 43 males) to create 36 random pairs. No prior personal relation was reported for all pairs. Hand dominance was self reported as the preferred hand for daily activities such as writing and eating. Participants gave informed written consent which was approved by the Institutional Review Board at Arizona State University and was in accordance with the Declaration of Helsinki.

### Experimental apparatus

A rigid U-shape sensorized long object (Fig. 1A) was used in the experiment. The object consisted of two grip handles mounted on a horizontal base. The center of mass was located at the mid-point of the base. The object’s weight was 1088 g and its height, length, and width were 185, 390 and 45 mm, respectively. Subjects were asked to grasp the handle(s) with all digits. Thumb and fingertips grasped the inner and outer sides of each handle, but participants could choose digit placement on two long graspable surfaces. Two 6-axis force/torques sensors measured forces and torques exerted by the thumb and all fingers on each handle (ATI Nano-25 SI-125–3; sampling frequency: 1 kHz). The tilt (error) of the device was shown to the participants by the bubble level placed in the mid-point of the horizontal base. We recorded the height and tilt of device using two infrared markers via a motion tracking system (Impulse, Phasespace Inc.; sampling frequency: 480 Hz).

### Experimental protocol

Participants grasped the object either individually with both hands (one on each handle, i.e., *Bimanual* condition; Fig. 1B), or cooperatively with another subject using one hand (i.e., *Dyadic* conditions; Fig. 1B). All participants received auditory cues through headphones to move the object upward or downward, and hold the object still within specific height ranges whose boundaries were denoted by rectangular bands placed on a vertical stand next to the center of the object.

Participants received four auditory cues. They positioned their hand(s) close to handle(s) before the beginning of each trial. When they heard first auditory cue (“lift up”), they grasped the handle and lifted the object at a natural speed while keeping it balanced until they reached the first target height range (45–55 mm). They held the object there until hearing the next auditory cue (“up”), then lifted the object to the second target height range (145–155 mm) and held it there until hearing the third auditory cue (“down”). Then they brought down the object to the first target height range and held it there until hearing the last auditory cue (“put down”), and replaced the object on the table. Participants were required to always follow the auditory cues to move between target heights. Importantly, we instructed participants to try their best to maintain the object balanced at all times when the object was in the air, so as to keep the bubble in the center of the level (equivalent to ± 1° error margin). Participants were also instructed to maintain the object at the height within the target range between auditory cues, while prioriziting the balance requirement. There was no instruction about the speed of their movement and participants were asked to collaborate with each other in the dyadic conditions. Verbal communication between a pair of participants was not allowed through the entire duration of the experiment.

For each pair of participants, there were a total of 6 experimental conditions which were performed in random order. Each experimental condition included 8 consecutive trials per, for a total of 48 trials (6 blocks × 8 trials). Each participant within a pair performed one bimanual condition individually (B1 and B2, therefore a total of two bimanual conditions). Additionally, four dyadic conditions were performed jointly (Fig. 1B). The dyadic conditions can be divided into two same-hand conditions that required coordination of either both dominant or non-dominant hands (DD and NN, respectively), and two different-hand conditions that require one partner using his/her dominant (right) hand and the other using his/her non-dominant (left) hand (DN and ND). The first and second letters of these acronyms denote the hand used by the participants who performed B1 and B2, respectively. In all experiments, the object was placed in the same way on the table, and the participants had to change their seating to form the desired spatical configuration. For DN and ND conditions, participants sat at the same side of the table (same side as bimanual conditions) where they were shoulder-to-shoulder with the left and right individuals using their left and right hands, respectively. In contrast, for DD and NN conditions, participants sat at the opposite side of the table facing each other. For all spatial configurations, the thumbs of both right or left hand were always located inside the U-shape object. This ensured consistency in the relative position of the object with respect to each arm to minimize the influence of biomechanical factors (e.g., supination/pronation). For this reason, we did not have the complete combination of hand dominance and pairing configuration.

### Data processing and experimental variables

Task performance has been quantified by object tilt as reported in our previous work^12^. In this study, our analysis focuses on the the behavioral variables that quantify the coordination of motor actions during the object balancing task (Fig. 1C). All data was synchronized and downsampled to 100 Hz, followed by low-pass filtering (cut-off 5 Hz). Note that each trial can be divided into multiple Dynamic and Static phases. The onset of the dynamic phase was the first time point at which the vertical position of the object center changed ± 5% relative to the previous vertical position averaged across 800 ms and stayed above that threshold for 600 ms. Similarly, the onset of the static phase was defined as the first time point after which the object vertical position computed over the past 600 ms remained within ± 5% relative to the vertical position averaged across the following 800 ms. For each trial, there were two dynamic phase segments and three static phase segments. In this investigation, we focused on the motor behavior in static phases (24 segments per dyad per condition), and the following metrics are all computed within each static phase segments.

#### Load force sharing

We define the net force excerted by one hand (thumb and fingers) in the opposite direction of gravity as the handle load force (positive upwards; *LF*_*1*_ and *LF*_*2*_ in Fig. 1A). They represent the amount of object weight sustained by the hand holding the corresponding handle. Thus, the sum of these two forces should always be approximately equal to the object weight. However, our task does not impose constraints on how the load can be distributed between the two handles, i.e., the load force sharing is a priori indeterminate. Therefore, we quantified the load sharing aspect of the coordination strategy as the average difference between two handle load forces, i.e., d*LF* = *LF*_*1*_ − *LF*_*2*_. This metric is zero if two hands share the load evenly. Note that for statistical reasons we define Handle 1 as the the right hand side in bimanual conditions. In contrast, Handle 1 was defined as the side held by the participant from B1 in all dyadic conditions (see arrows in Fig. 1C).

#### Internal force

For each handle, we computed the net force exerted in the direction normal to the grasp surface, with the positive direction pointing inwards of the object (*IF*_*1*_ and *IF*_*2*_ in Fig. 1A). These two forces should always be approximately equal to each other for the object to remain stationary horizontally. However, the magnitude of these forces can take any value in either inward or outward directions without compromising the requirement of keeping the object still. Therefore, we defined the internal force as *IF* = (*IF*_*1*_ + *IF*_*2*_)/2, averaged within each static phase segment. This metric is positive or negative if the two hands are pushing or pulling against each other, respectively.

#### In-hand moment sharing

In the object balancing task, if the load forces were not shared evenly or the points of normal force application were not perferctly aligned horizontally between two handles, there would be a moment that needs to be compensated to prevent object rotation. This was achieved by each hand excerting an in-hand moment with supination/pronation (*M*_*1*_*, M*_*2*_ in Fig. 1A). This moment can be calculated about the center of each handle using load force, normal force and center of pressure measured at each grasp surface of that handle ^28^. Similar to load force sharing, a net compensatory moment can be in theory achieved through an infinite number of combinations of in-hand moments d*M* = *M*_*1*_ − *M*_*2*_. Importantly, however, this metric was strongly correlated with load force sharing due to the overall task constraint that requires zero net moment for the object to remain balanced (e.g., Fig. 1C; See Supplementary Fig. 1). Therefore, we do not report this metric, and we only focus on load force sharing.

#### Handle vertical velocity

We take the first derivative of handle vertical positions to obtain vertical velocities (*V*_*1*_*, V*_*2*_ in Fig. 1A). We did not quantify horizontal movement because the main task goals were minimizing object tilt and maintaining object height, which are both accomplished by handle vertical movements. There are two metrics that we derive from handle vertical velocities. First, we compute the mean absolute value (MAV) of the velocities to quantify the extent to which each participating hand moves. Unlike other metrics in this paper, the MAV of velocities are computed for each hand. This is because the movement of each hand can be theoritically independently controlled. In contrast, both load force sharing and internal force are pair-wise metrics because two handles were not independent from each other due to force equilibrim constraints.

#### Delay between handle movements

We quantify the temporal aspect of the movement coordination using cross-correlation between *V*_*1*_ and *V*_*2*_. The lag corresponding to the largest positive peak within ± 500 ms of the normalized cross-correlation function were identified. Note that normalized cross-correlation was used so that the the magnitude of the velocities do not contribute to the results. We only quantify the absolute value of this lag, which represent the synchronization between two hands. Similar to force metrics, this delay is also a pair-wise metric.

### Statistical analyses

All metrics were first averaged across 24 static phase segments within each condition for each pair (or each hand). Statistical analysis for each metric began with comparisons between bimanual conditions (B1 and B2) and followed by linear mixed model analysis within dyadic conditions (DN, ND, DD and NN). The methods we used for pair-wise metrics (load force sharing, internal force, delay between handle movements) and subject-wise metric (handle velocity) were different, and they are described below separately.

#### Pair-wise metrics

For Bimanual conditions, we first use t-tests to validate that participants who were assigned to B1 and B2 were from the same population. This justifies the linear mixed models for dyadic conditions which consider four different pairing scenarios with respect to the participant from B1 only, as modeling with respect to the participant from B2 would yield similar results. For dyadic conditions, we used mixed effect models with respect to participants from B1 in the form of

$${y}_{n}= {b}_{0}+{b}_{1}{P}_{H}+{b}_{2}{P}_{C}+{b}_{3}{P}_{HC}+{b}_{p}$$

where ${y}_{n}$ is the behavioral metric, ${P}_{H}$ and ${P}_{C}$ are fixed factors representing Hand (used by participants from B1, two levels: left and right) and pairing Configuration (two levels: face-to-face same-hand and side-by-side different-hand).${P}_{HC}$ is the interaction between the fixed factors. ${b}_{0}$ is the constant intercept, ${b}_{1}$, ${b}_{2}$ and ${b}_{3}$ are the coefficients for fixed factors. ${b}_{p}$ is the pair-wise random effect. Post-hoc comparisons was performed with paired t-tests and Bonferroni corrections. Additionally, we also assessed the within-condition variability for load force sharing and internal foce metrics. This was achieved by paired comparison of the absolute deviation from condition mean between six conditions using non-parametric Wilcoxon Signed Ranks tests.

#### Subject-wise metric

For Bimanual conditions, we first use two-way mixed ANOVA (Hand × Group) to examine the effect of hand dominance and validate that participants who are assigned to B1 and B2 were from the same population. For dyadic conditions, we used a similar mixed effect model used for all subjects:

$${y}_{n}= {b}_{0}+{b}_{1}{P}_{H}+{b}_{2}{P}_{C}+{b}_{3}{P}_{HC}+{b}_{s}$$

where ${y}_{n}$ is the behavioral metric, ${P}_{H}$ and ${P}_{C}$ are fixed factors representing Hand (used by participants, two levels) and pairing Configuration (two levels: face-to-face same-hand and side-by-side different-hand).${P}_{HC}$ is the interaction between the fixed factors. ${b}_{0}$ is the constant intercept, ${b}_{1}$, ${b}_{2}$ and ${b}_{3}$ are the coefficients for fixed factors. ${b}_{s}$ is the subject-wise random effect. Post-hoc comparisons was performed with paired t-tests and Bonferroni corrections.

## Supplementary Information

Supplementary Figures.

## Data Availability

The datasets generated during and/or analysed during the current study are available from the corresponding author on reasonable request.
