# Supplementary material for: Inter-personal motor interaction is facilitated by hand pairing
Source: Sci Rep. 2022 Jan 11;12:545. doi: 10.1038/s41598-021-04595-9 (PMC8752769; doi:10.1038/s41598-021-04595-9)
Supplement: Supplementary file 1 — Supplementary Figures. [file 41598_2021_4595_MOESM1_ESM.docx]

**Supplementary materials**

**1. Correlation between Load Force sharing and In-Hand Moment Sharing.**


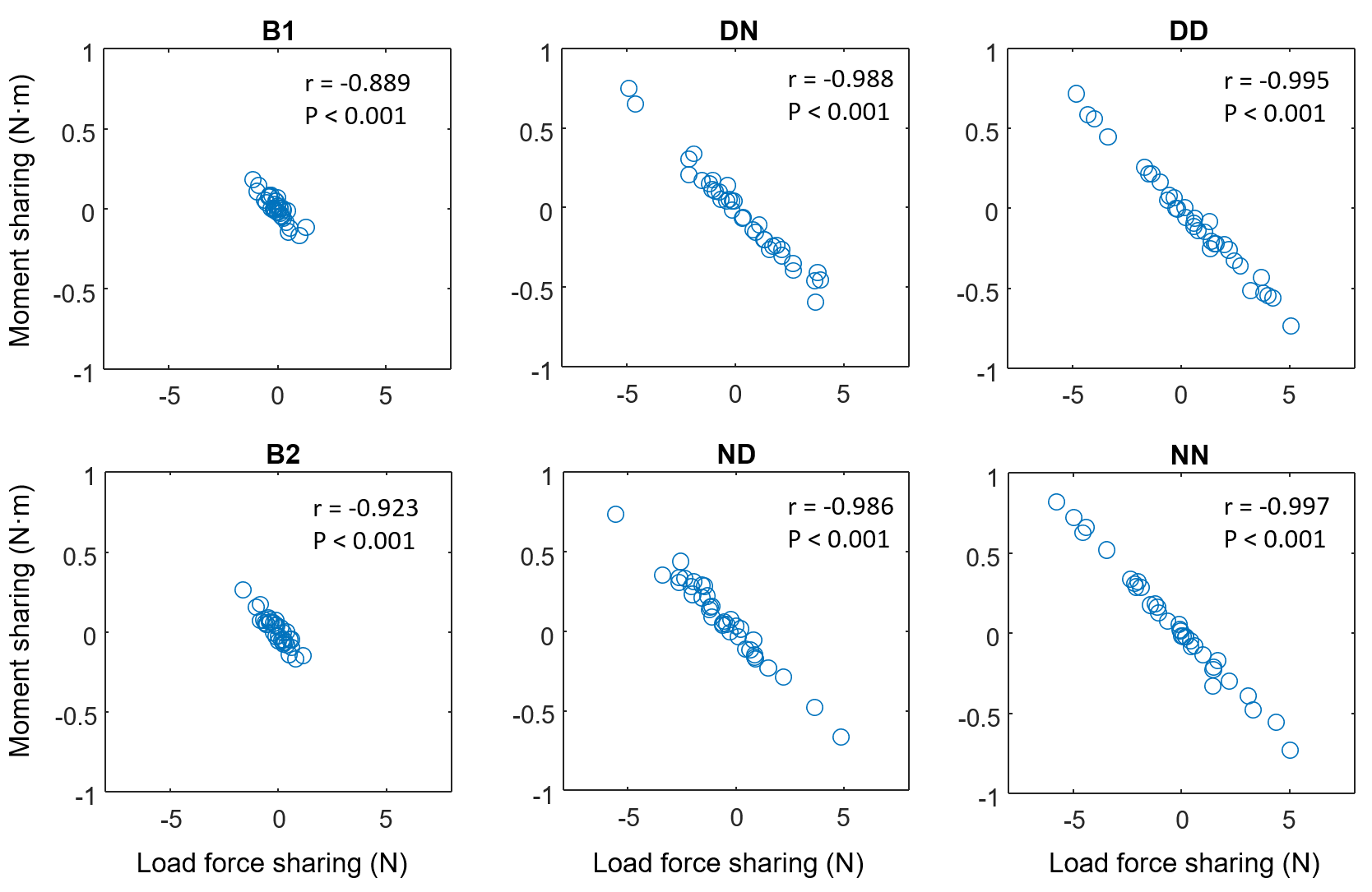


**Supplementary Figure S1**. We validated the relation between Load Force sharing and In-Hand Moment Sharing induced by the task requirement of balancing the object. The Pearson’s r-value and the p-values are shown for each relation. Each data point is from individual participants in bimanual conditions (B1 and B2) or a dyad in dyadic conditions (DN, ND, DD, and NN). In this study we only report the load force sharing between two hands because the sharing patterns of in-hand moment is highly correlated with the load force sharing patterns.

**2. Within-condition variability of load force sharing.**


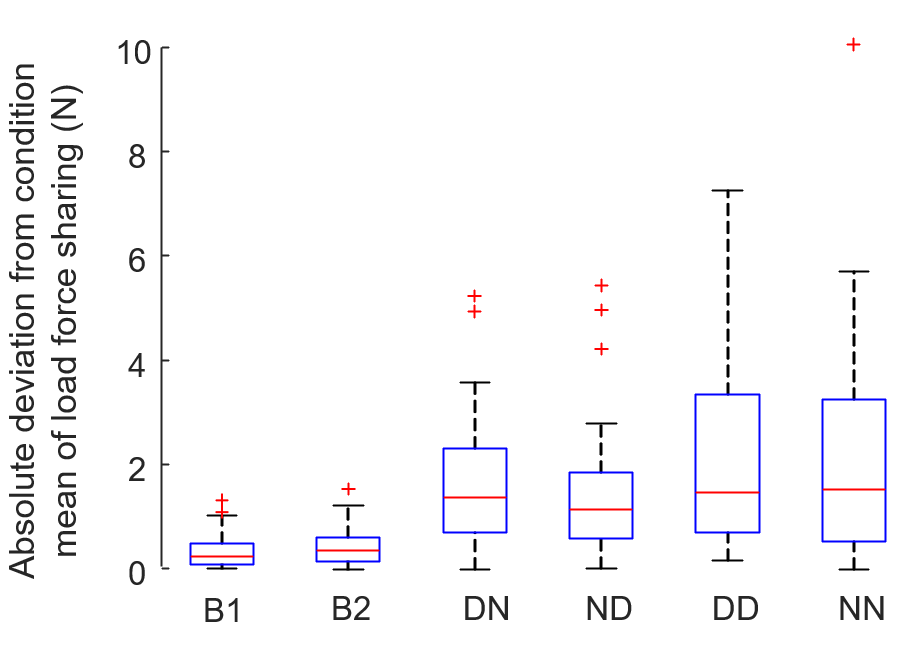


**Supplementary Figure S2.** We computed the absolute deviation from the condition mean of load force sharing to quantify the within-condition variability of this metric. Boxplots show median values as red lines, with 25 and 75 percentiles depicted as blue boxes. The whiskers extend to most extreme points that are not potential outliers (i.e., red crosses). The median values of the absolute deviations were: 0.257 N, 0.357 N, 1.382 N, 1.144 N, 1.490 N, and 1.536 N for B1, B2, DN, ND, DD, and NN conditions, respectively. We used pair-wise Wilcoxon Signed Ranks tests with Bonferroni corrections. For all comparisons between one Bimanual condition and one Dyadic condition, we found statistically significant differences (p < 0.001). p-values were not statistically significant for comparisons between two Bimanual conditions or between any pair of Dyadic conditions.

**3. Within condition variability of internal forces.**


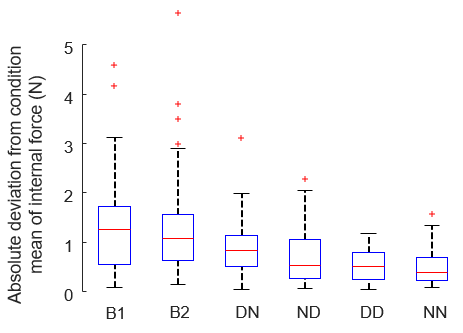


**Supplementary Figure S3.** We computed the absolute deviation from the condition mean of internal force to quantify the within-condition variability of this metric. Data are shown in the same format as Supplementary Figure 1. The median values of the absolute deviations were: 1.257 N, 1.066 N, 0.828 N, 0.527 N, 0.502 N, and 0.392 N for B1, B2, DN, ND, DD, and NN conditions, respectively. We used pair-wise Wilcoxon Signed Ranks tests with Bonferroni corrections. Variability in bimanual conditions were significantly larger than those in same-hand conditions (DD and NN, p < 0.001), and there was no difference between B1 and B2 nor DD and NN. Although different-hand conditions were characterized by medium variability, we did not find significant differences in most of the comparisons, except between B1 and ND (p = 0.003; some comparisons were significantly different at p < 0.05 but were not after Bonferroni corrections).

**4. Correlation between internal force and lag between movements.**


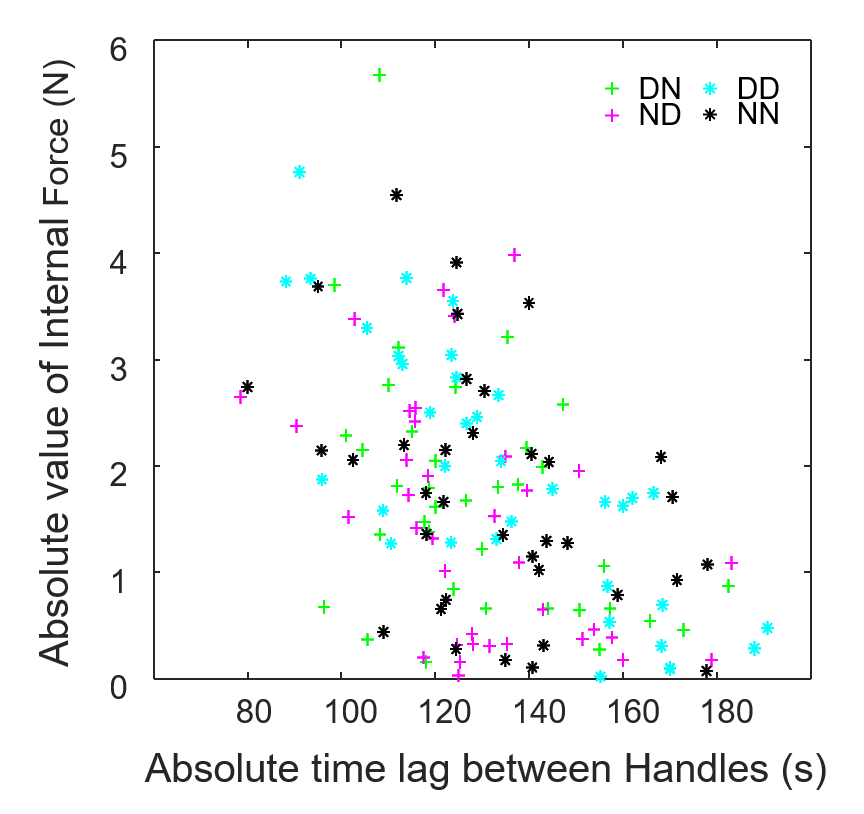


**Supplementary Figure S4.** Each data point represents one pair from one condition. In addition to the correlation coefficient obtained from analysis of all samples, we also computed the correlation coefficient within each condition. DN: r = –0.423, p = 0.010; ND: r = –0.449, p = 0.006; DD: r = –0.797, p < 0.001; NN: r = –0.406, p = 0.014.

**5. Distribution of Load Force and In-Hand Moment.**


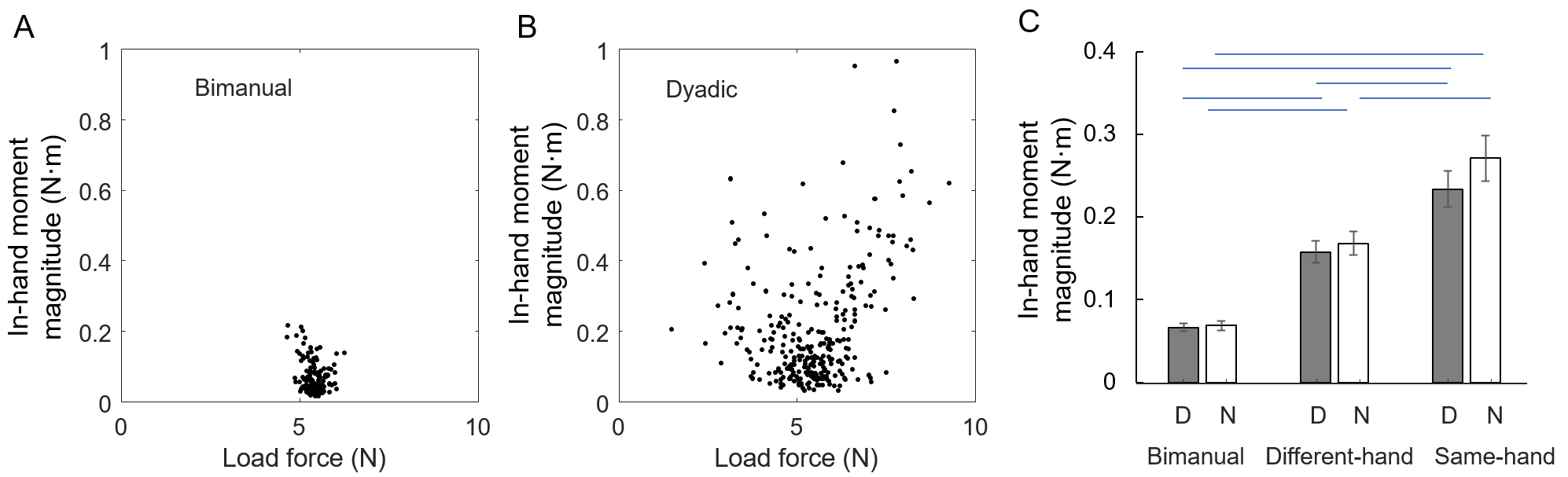


**Supplementary Figure S5.** We computed the magnitude of the In-hand moment for each hand involved in the bimanual and dyadic conditions. They are plotted with respect to the Load force associated with the same hand (Panel A and B for Bimanual and Dyadic conditions, respectively). It can be observed that Load forces were close to 5.3 N for each hand as two hands share the weight of the object symmetrically. In contrast, Load forces were in a much wider range as two hands share the weight more asymmetrically in dyadic conditions, which also led to higher magnitude of In-hand moment to compensate. Two-way repeated measure ANOVA (Hand × Pairing) revealed significant effect of Pairing (p < 0.001). Post-hoc t-test revealed significant differences between Pairing configurations (p < 0.001, Panel C), with DD and NN condition showing largest In-hand moment magnitude whereas Bimanual conditions showing the smallest.
